# Pre-sliding technique to improve femoral neck system against the shortening: a retrospective cohort study

**DOI:** 10.1186/s12891-024-07391-4

**Published:** 2024-04-16

**Authors:** Dongze Lin, Yaqian Liang, Peisheng Chen, Shunze Zheng, Fengfei Lin

**Affiliations:** 1 Department of Orthopaedics, Fuzhou Second General Hospital, Fujian Provincial Clinical Medical Research Center for First Aid and Rehabilitation in Orthopaedic Trauma, Fuzhou, 350007 China; 2 School of Clinical Medicine, Fujian Medical University, Fuzhou, 350007 China

**Keywords:** Femoral neck fracture, Fracture fixation, Internal, Femoral neck system, Femoral neck shortening, Pre-sliding

## Abstract

**Objective:**

To investigate the efficacy of using pre-sliding technique to prevent postoperative shortening of displaced femoral neck fracture fixed with femoral neck system (FNS).

**Methods:**

Retrospective analysis of 110 cases of displaced femoral neck fracture treated with femoral neck system from September 2019 to November 2022 in our center, which were divided into 56 cases in the pre-sliding group and 54 cases in the traditional group. The baseline data such as gender, age, side, mechanism of injury, fracture type, operation time, intraoperative bleeding were recorded and compared between the two groups, and the quality of fracture reduction, shortening distance, Tip Apex Distance (TAD), union time, Harris score of the hip were also compared between the two groups.

**Results:**

The TAD value of the pre-sliding group was smaller than that of the traditional group, and the difference was statistically significant (*P* < 0.001). The shortening distance in both groups on postoperative day 1 was smaller in the pre-sliding group than in the traditional group, but the difference was not statistically significant (*P* = 0.07), and the shortening distance was smaller than in the traditional group at 1, 3, 6, and 12 months postoperatively, and the difference was statistically significant (all *P* < 0.001). Of the 110 cases, 34 (30.9%) had moderate or severe shortening, of which 24 (44.4%) were in the traditional group and 10 (17.9%) in the pre-sliding group, and the difference was statistically significant (*P* < 0.001), and the Harris score at 1 year, which was higher in the pre-sliding group than in the traditional group, and the difference between the two groups was statistically significant (*P* < 0.001). There was no statistically significant difference in the comparison of baseline data such as gender, age, side, mechanism of injury, fracture type, operation time, intraoperative bleeding, and quality of reduction between the two groups (all *P* > 0.05), and no statistically significant difference in fracture healing time between the two groups (*P* = 0.113).

**Conclusion:**

The use of the pre-sliding technique of displaced femoral neck fracture fixed with FNS reduces the incidence of moderate and severe shortening, improves the postoperative TAD value, and improves the hip function scores, with a satisfactory midterm efficacy.

More than 6 million people are expected to suffer hip fractures globally by 2050 [1–3], and femoral neck fracture is the most common fracture of the hip, accounting for approximately 53% of hip fractures [1]. The Garden classification is widely used in clinical practice to categorize femoral neck fractures, which are divided into 4 types, of which Garden I and II are classified as nondisplaced fractures, and Garden III and IV are displaced fractures [3]. For patients with femoral neck fracture in young or middle, reduction and fixation is the preferred surgical option, but the complications after internal fixation of femoral neck fracture are still as high as 20-30% [4–7], and the complication rate is even higher in displaced fracture, which is known as “unresolved” fracture, and the new internal fixation has brought more choices to orthopedic surgeons and new problems at the same time.

Femoral neck system (FNS) was marketed in 2017, and its biomechanical test shows that compared with the traditional method, it can better resistance to valgus and rotation [8], and can be implanted through a small incision(about 4 cm) at the same time. The sliding distance reserved for the FNS allows for axial compression of the femoral neck fracture after fixation and promotes fracture union. However, in displaced fractures, especially in patients with cortical comminution, the contact area between the fractures is insufficient to develop an effective anti-shortening mechanism, and severe shortening of the femoral neck can occur early after FNS fixation.

Femoral neck shortening is a common early complication, and the incidence of femoral neck shortening after FNS fixation reported in the literature is 28-39.1%, while the incidence of shortening more than 5 mm is as high as 43.7% of displaced femoral neck fractures, with an average distance of 4.9–8.4 mm [9, 10]. The resulting shortening of the femoral neck affects the abductor arm of the hip joint, causing a reduction in the eccentric distance and alterations in hip function and gait. A shortening of more than 5 mm has a significant impact on the quality of life of patients [11]. What measures can be used to prevent shortening when fixing displaced femoral neck fractures with FNS?

Our center has been using the pre-sliding technique in FNS fixation of displaced femoral neck fractures since September 2019 in order to improve FNS resistance to shortening. The aims of this retrospective cohort study are: to understand the characteristics of postoperative femoral neck shortening in FNS displaced fractures, the effect of femoral neck shortening on hip joint function, and the effectiveness of the pre-sliding technique in preventing postoperative shortening in displaced femoral neck fractures.

## Materials and methods

### Study design

This is a retrospective cohort study that was reported in accordance with the STROBE statement [12]. The study adhered to the Declaration of Helsinki as revised in 2013, and was conducted after approval from the Ethics Committee of Hospital (No. 2,021,185). A retrospective cohort study was carried out on young and middle-aged patients who had suffered displaced femoral neck fractures (Garden type III/IV) and undergone closed reduction FNS fixation at our hospital between September 2019 and November 2022, and were divided into the pre-sliding group(use of the pre-sliding) and traditional group.

### Inclusion and exclusion criteria

Patients were eligible for inclusion according to the following criteria: patients who received a diagnosis of femoral neck fracture at our center based on X-ray and were classified as displaced(Garden III and IV) [13]; the FNS (Johnson & Johnson, USA) was employed. Age 18 or older, participants were followed up for at least 12 months. Cases with pre-injury co-morbidities affecting the function of the hip (such as hip dysplasia on the affected side and osteoarthritis) as well as those with incomplete follow-up data, multiple fractures, pathological fractures, poor reduction(Garden Index III and IV), or those who underwent open reduction were excluded from the analysis.

### Surgical procedure

**Fig. 1:**
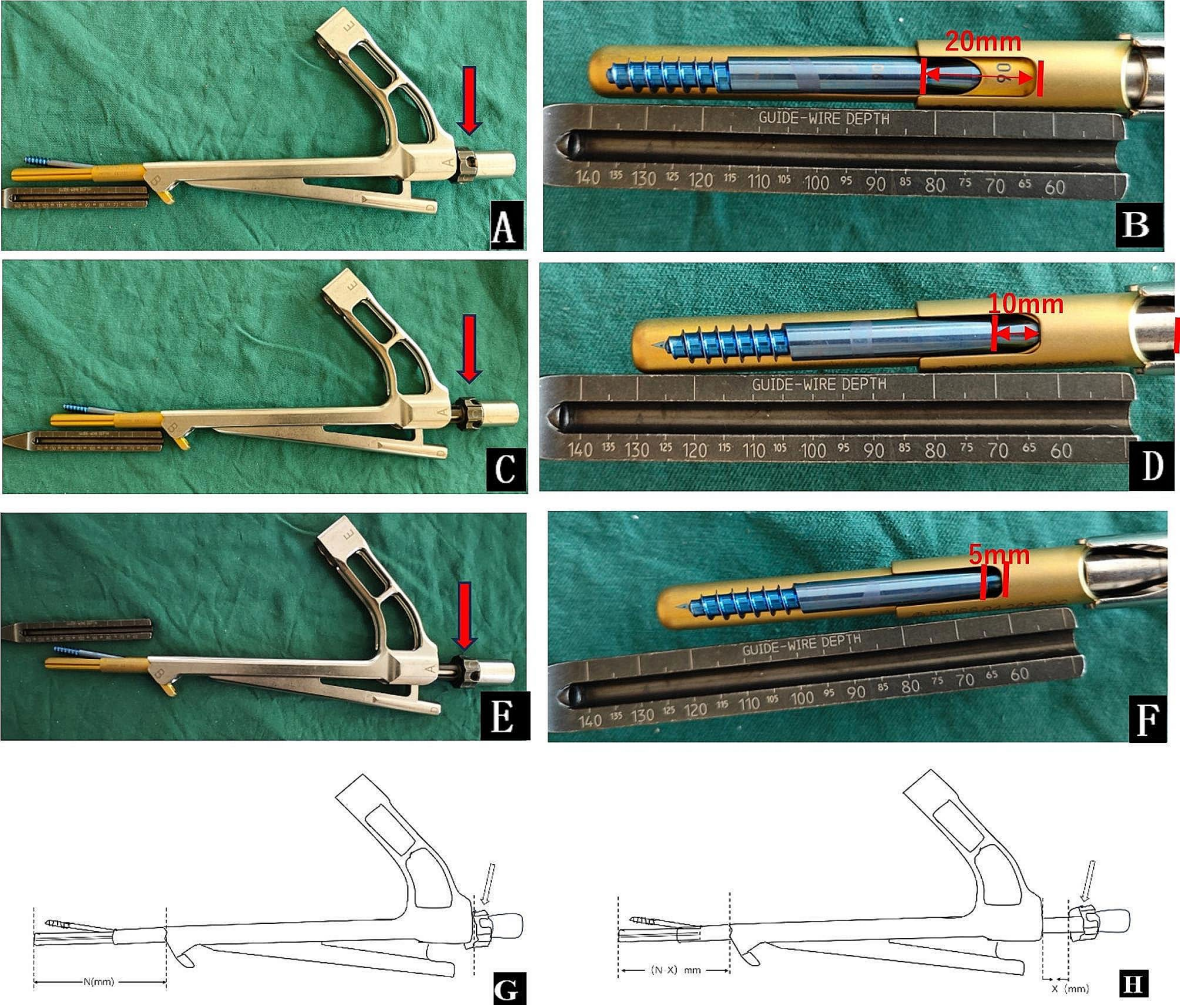
Schematic for Pre-sliding (**A**, **B**) According to the FNS operation guide, the handle at the red arrow is screwed to the end clockwise, retaining the maximum sliding distance (20 mm), at this time, the effective length of the FNS is 90 mm (**C**, **D**) The handle is screwed to the end clockwise and then unscrewed counterclockwise by a distance of about 10 mm (red arrow), at this time, the sliding distance is retained as 10 mm, and the effective length of the FNS is 80 mm (**E**, **F**) The handle is screwed to the end clockwise and then unscrewed counterclockwise by a distance of about 15 mm (red arrow), which is the maximum pre-sliding distance, at this time, the sliding distance is retained as 5 mm, and the effective length of the FNS is 75 mm G, H Schematic before (**G**) and after(**H**) pre-sliding, pre-sliding occurs by rotating the nut (white arrow), N represents the length of the FNS before pre-slip, X represents the pre-slip distance, and (N-X) represents the length of the FNS after pre-sliding

After general anesthesia and sterilize. Along the anterior-superior of the femoral neck, insert two Kirschner pins, not exceeding the fracture line. Closed reduction is performed, and intraoperative fluoroscopy is utilized to assess the quality of the reduction. When the reduction is deemed satisfactory, the two pre-positioned Kirschner’s pins are inserted into the femoral head for temporary immobilization. The incision is made following the FNS manufacturer’s guidelines, and under fluoroscopy, the guide pin is drilled approximately 5 mm below the cartilage of the femoral head through a 130° guide. The depth of the guide pin embedded in the femoral head was measured. Based on the measured depth of the guide pin embedded in the femoral head, the pre-sliding group performing the pre-sliding maneuver (Fig. 1) used a structure that was two sizes larger than the measured value (10 mm larger than the size recommended by the manufacturer’s guidelines), while the traditional group chose the structure recommended by the manufacturer’s guidelines based on the measured value. For example, if the measured value is 95 mm, the pre-slip group selects a 105 mm long bolt, and in the traditional group a 95 mm long bolt is recommended according to the manufacturer’s guide. Install the bolt, locking screw, and anti-rotation screw in that order. Fluoroscopy is performed again to confirm screw position and fracture repositioning.

### Perioperative management and follow-up

All patients received intravenous cefazolin sodium half an hour before and 24 h after surgery to prevent infection. Depending on fracture healing, partial weight-bearing (20–30 kg) on the affected limb was instructed at 6 to 8 weeks postoperatively, with a gradual transition to full weight-bearing. Bilateral anteroposterior and lateral hip radiographs were taken after admission and on the first postoperative day, and patients were notified to come to the outpatient clinic for follow-up radiographs at 1, 3, 6, and 12 months postoperatively (notified in advance by phone or text message), for hip function assessment, and for registration (Zheng was in charge of registration, entry, and storage of data).

### Data collection and measurement

Baseline data included: Age, gender, body mass index, mechanism of injury, side of injury, Garden classification, Pauwels classification, cortical comminution, use of pre-sliding technique or not, operative time and bleeding. Quality of fracture reduction: Garden index [14]: The quality of resetting can be divided into 4 grades: grade I: 160° antero-posterior and 180° lateral; grade II: 155° to 160° antero-posterior and 180° lateral; grade III: 150° to 155° antero-posterior or > 180° lateral; grade IV: <150° antero-posterior and > 180° lateral. The higher the grade, the poorer the quality of the repositioning.

**Fig. 2:**
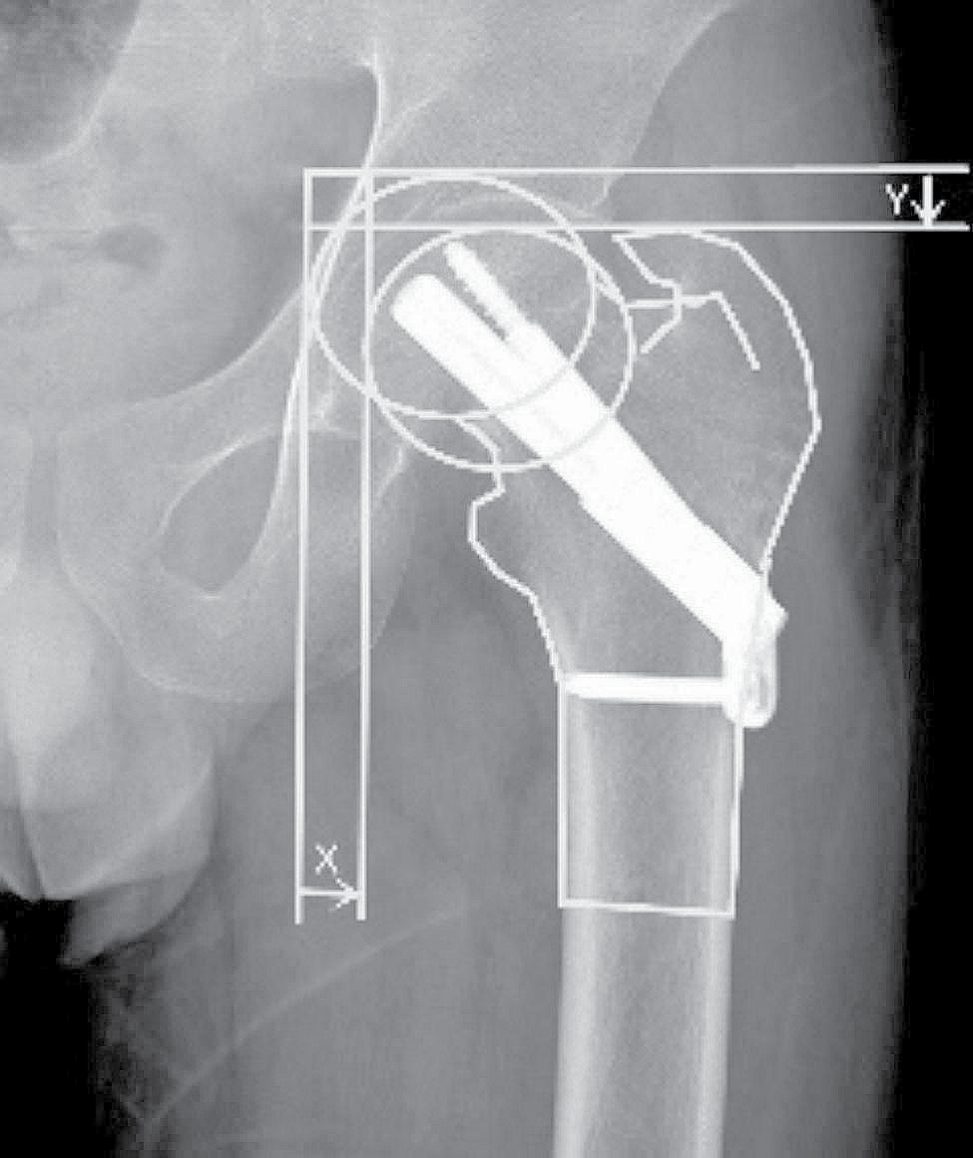
Mirror the contralateral hip to the affected hip and measure the horizontal shortening of the femoral head set to the X-axis, the vertical shortening of the femoral head set to the Y-axis, and the upward shortening distance of the femoral neck axis Z = Ysin(θ) + Xcos(θ) (θ is the angle between the Y-axis and the femoral neck axis)

**Fig. 3:**
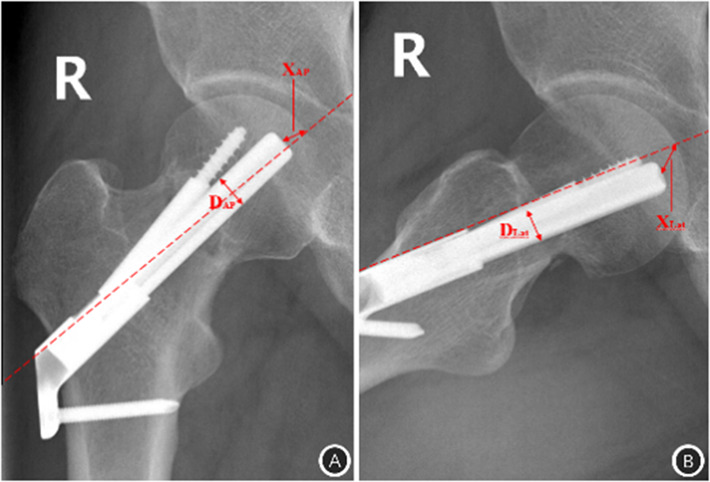
TAD measurement after FNS A Hip anteroposterior position B Hip lateral position The long dotted line (red) indicates the femoral head and neck mid-axis, the distance from the tip of the bolt (Tip) to the apex of the femoral head and neck mid-axis and femoral head-articular surface junction (Apex) was measured on the anteroposterior and lateral side respectively, the distance between the anteroposterior and lateral side (Xap), the distance between the anterior and posterior side (Xlat), and the widths of anteroposterior and lateral side bolt measurements (Dap, Dlat), and the amplification factor was corrected with the actual bolt width (Dtrue) (TAD = Xap×(Dtrue/Dap) + Xlat×(Dtrue/Dlat))

Primary outcome measures: Degree of shortening: Zlowodzki method [15]: The degree of femoral neck shortening was classified into mild shortening (< 5 mm), moderate shortening (5–10 mm) and severe shortening (> 10 mm) (Fig. 2). Tip to Apex Distance (TAD): In the immediate postoperative period, at the anteroposterior and lateral radiograph, the distance from the tip of the screw (tip) to the apex of the junction of the mid-axis of the femoral head neck and the articular surface of the femoral head (apex) was measured. Based on the concept of TAD for the power hip screw proposed by Baumgaertner et al. in 1995 [16], the distance between the tip of the screw (Tip) and the apex of the junction of the medial axis of the femoral head neck and the articular surface of the femoral head (Xap), the lateral position distance (Xlat), and correcting the magnification factor of the X-ray with the actual width of the bolt (Dtrue) and the measured width of the anterior-posterior and lateral position bolts (Dap, Dlat), and the sum is the TAD value (TAD = Xap×(Dtrue/Dap) + Xlat× (Dtrue/Dlat) (Fig. 3).

Secondary outcome measures: Fracture union assessment criteria [17] : no obvious percussion pain in the hip joint and lower limb on the operated side. Radiograph or CT showed a blurred fracture line and continuous cancellous bone trabeculae crossing the original fracture line. Functional score: Harris hip function score [18]: assessed from 4 aspects of joint mobility, function, pain and deformity, with a total score of 100 points; excellent 90–100 points, good 80–89 points, acceptable 70–79 points, and poor 70 points or less. Complications: incidence of postoperative complications [6] (assessed by two attending surgeons), including (1) deep incision infection; (2) implant cutout (implant passing through the femoral head into the joint); (3) implant displacement: Displacement of the bone phase relative to the implant (no femoral head cutout); (4) implant failure (fracture/bending); (5) delayed healing or non-healing; and (6) femoral head necrosis; (7) secondary hip arthroplasty.

### Statistics

Sample size calculation: According to the incidence rate of moderate and severe shortening following fixed femoral neck fracture with FNS was about 30% [9]. The pre-sliding group was predicted to have an incidence rate of about 15%. A two-sided test was conducted with α = 0.05 and β = 0. 1. With a test efficacy degree of 1- β = 90% and a 1:1 ratio of sample sizes between the two groups, the minimum sample size for each group is determined to be 37 cases. Considering a dislodgement rate of 10%, the minimum sample size for each group is subsequently increased to 41 cases. Statistical analyses were performed using SPSS 26.0 software (IBM SPSS, USA). Count data, sex, smoking/alcohol use, mechanism of injury, side of injury, Garden typing, Pauwels typing, cortical comminution of the amputated end, quality of resurfacing, and shortening grading at 1 year postoperatively were compared using the χ2 test. For the measurement data, the Shapiro-Wilk test was used to determine whether it met the normal distribution, and its age and BMI were normal data and variance chi-squared, expressed as (x ®± s), and two independent samples t-tests were used to compare the two groups of patients; where the measurement data for the time of surgery, amount of intraoperative bleeding, TAD value, time of fracture healing, 1-year postoperative Harris score, and shortening distance not conforming to normal distribution were expressed as M(Q1,Q3), and comparisons between the two groups were made using the Mann-Whitney U test. *P* < 0.05 was considered statistically significant.

## Results

There were 348 cases of femoral neck fractures internally fixed with FNS, of which 137 (39.4%) patients with displaced femoral neck fractures, 10 cases with incomplete follow-up information (patients did not follow up and could not be contacted by phone), 2 cases of open reduction, 12cases of poor reduction, and 3 cases of multiple fractures were excluded, a total of 110 cases were included in this study, among which 56 cases were in the pre-sliding group and 54 cases were in the traditional group, with a follow-up time of mean (SD) 19.4 ± 3.9 months (12–26 months). There was no statistically significant difference in the comparison of baseline data such as gender, age, side of injury, mechanism of injury, Classification of Fractures, and cortical comminution (Table 1).

**Table 1:** Comparison of baseline data and Surgical data between traditional group and pre-sliding group

Groups		Traditional group (*n* = 54)	Pre-sliding group (*n* = 56)	*P* value
Age	($\stackrel{-}{\text{x}}$±*s*)	47.5 ± 13.1	50.38 ± 12.8	0.249
Gender	Male	38(70.4%)	34(60.7%)	0.287
	Female	16(29.6%)	22(39.3%)	
BMI	($\stackrel{-}{\text{x}}$±*s*)	23.9 ± 2.5	23.2 ± 2.5	0.155
Smoking	No	48(88.9%)	53(94.6%)	0.271
	Yes	6(11.1%)	3(3.4%)	
Drinking	No	48(88.9%)	53(94.6%)	0.271
	Yes	6(11.1%)	3(3.4%)	
Mechanism of injury	Low energy	19(35.2%)	14(25.0%)	0.244
	High energy	35(64.8%)	42(75.0%)	
Side of Injury	Left	20(37.0%)	25(44.6%)	0.417
	Right	34(63.0%)	31(55.4%)	
Garden Classification	Garden III	22(40.7%)	17(30.4%)	0.255
	Garden IV	32(59.3%)	39(69.6%)	
Pauwels Classification	Pauwels I	22(40.7%)	16(28.6%)	0.284
	Pauwels II	12(22.3%)	19(33.9%)	
	Pauwels III	20(37.0%)	21(37.5%)	
Cortical Comminution	No	18(33.3%)	21(37.5%)	0.648
	Yes	36(66.7%)	35(62.5%)	
Time from injury to surgery (days)	M(Q1,Q3)	3.0(2.0,4.0)	3.0(3.0,5.0)	0.282
Surgical time (min)	M(Q1,Q3)	61.5(55.0,71.2)	52.0(40.0,68.0)	0.003
Bleeding (ml)	M(Q1,Q3)	50.0(20.0,50.0)	42.5(20.0,50.0)	0.317
Reset grading	Garden index I	52(96.3%)	51(91.1%)	0.262
	Garden index II	2(3.7%)	5(8.9%)	
Healing time (weeks)	M(Q1,Q3)	8.0(6.0,10.0)	6.0(6.0,10.0)	0.113
Harris score	M(Q1,Q3)	84.1(79.9, 89.0)	91.5(88.05, 93.7)	<0.001

### Surgical details

Closed repositioning with 1-hole FNS fixation was performed in all cases. Comparison of time from injury to surgery, time from skin incision to suture, bleeding, and quality of repositioning between the two groups is shown in Table 1, and the difference in surgical details between the two groups was not statistically significant (all *P* > 0.05).

### Primary outcome data

**Fig. 4:**
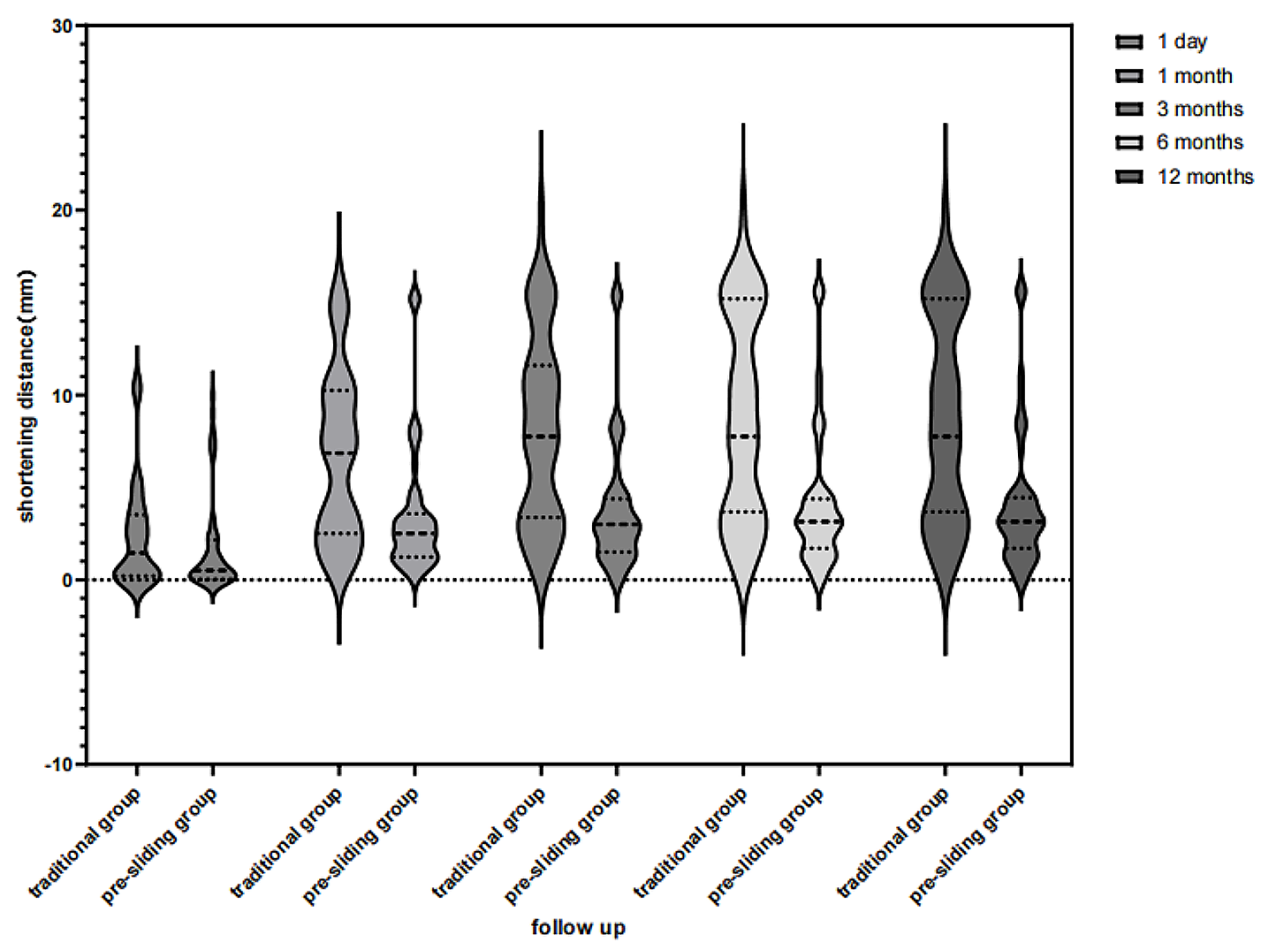
Shortened distance between the two groups at follow-up

**Fig. 5:**
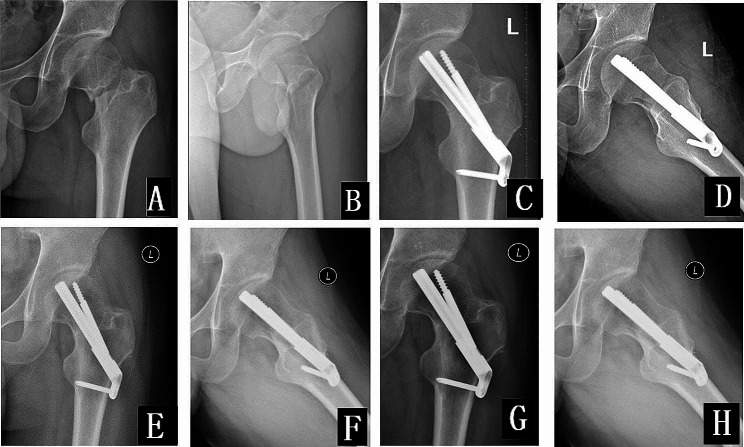
24-year-old male with a left femoral neck fracture due to a fall from a height (**A**, **B**) antero-posterior and lateral view of the hip, displaced femoral neck fracture, cortical comminution of the medial-posterior (**C**, **D**) antero-posterior and lateral view on the first postoperative day, and using the pre-sliding technique, with a pre-sliding of approximately 10 mm, and the fracture is good reduction (**E**, **F**) antero-posterior and lateral view at 3 months postoperatively, with the fracture healed, with internal fixation in place, and with no occurrence of moderate or severe shortening of the femoral neck (**G**, **H**) antero-posterior and lateral view at 1 year and 3 months postoperatively, with no necrosis of the femoral head, and with no persistent shortening of the femoral neck

The Tip Apex Distance (TAD) was measured on radiographs on the first postoperative day, and the value in the pre-sliding group was smaller than that in the traditional group, with a statistically significant difference (*P* < 0.001). Comparison of postoperative shortening distances as shown in Table 2, the shortening distances of both groups on postoperative day 1 were smaller in the pre-sliding group than in the traditional group, but the difference was not statistically significant (*P* = 0.07), and the shortening distances at 1, 3, 6, and 12 months postoperatively were smaller than those in the traditional group, and the differences were statistically significant (all *P* < 0.001). According to the shortening grading, 34 (30.9%) of 110 cases (shortening ≥ 5 mm) had moderate to severe shortening, including 24 (44.44%) cases in the traditional group and 10(17.9%, 10/56) cases in the pre-sliding group, and the difference was statistically significant (*P* < 0.001). Shortened distance between the two groups at follow-up(Fig. 4) and a typical case of pre-sliding group(Fig. 5).

**Table 2:** Comparison of TAD and Shortening between traditional group and pre-sliding group

Groups		Traditional group(*n* = 54)	Pre-sliding group(*n* = 56)	*P* value
TAD value(cm)	M(Q1,Q3)	2.5(1.8, 3.4)	1.3(0.9, 1.6)	<0.001
Shortening distance (1 day, mm)	M(Q1,Q3)	1.5(0.2, 3.5)	0.5(0.1, 2.2)	0.07
Shortening distance (1 month, mm)	M(Q1,Q3)	6.9(2.5, 10.3)	2.5(1.2, 3.6)	<0.001
Shortening distance (3 months, mm)	M(Q1,Q3)	7.8(3.4, 11.6)	3.0(1.5, 4.4)	<0.001
Shortening distance (6 months, mm)	M(Q1,Q3)	7.8(3.7, 15.2)	3.2(1.7, 4.4)	<0.001
Shortening distance (1 year, mm)	M(Q1,Q3)	7.8(3.7, 15.2)	3.2(1.7, 4.5)	<0.001
Shortening grading (1 year)	Mild(<5 mm)	30(55.6%)	46(82.2%)	<0.001
	Moderate(5–10 mm)	10(18.5%)	5(8.9%)	
	Severe(>10 mm)	14(25.9%)	5(8.9%)	

### Secondary outcome data

The fracture healing time between the two groups was not statistically significant (*p* = 0.113); the Harris hip function score at 1 year after surgery between the two groups was statistically significant (*p* < 0.001) (Table 1).

### Complications

In the traditional group, there was one case of implant cut-out where the implant passed through the femoral head into the joint, and in the pre-sliding group, two cases were reported. Additionally, implant displacement occurred in one case in the traditional group due to displacement of the bone phase in response to the implant, without femoral head cut-out, while no cases were reported in the pre-sliding group. Two cases of delayed healing were observed in each group, but they eventually healed after prolonged observation. Both groups experienced three cases of femoral head necrosis; the traditional group had one case of combined cut-out and the pre-sliding group had two cases occur 14–18 months post-surgery. All five cases underwent total hip arthroplasty.

## Discussion

Displaced femoral neck fractures are unstable and frequently involve cortical comminution. After fixation, complications like nonunion, femoral head necrosis, and femoral neck shortening can occur and prove to be a substantial burden for patients. Despite bringing hope, the new internal fixation(FNS) also gives rise to new issues. Many patients with displaced femoral neck fractures experience moderate and severe shortening of the femoral neck in the early postoperative, leading to a poor hip function. Our observation suggests that employing the pre-sliding enhances the TAD value and resistance to shortening, and reducing the incidence of femoral neck shortening in the postoperative.

### Impact of femoral neck shortening on hip function

As the functional demands of femoral neck fractures increase, postoperative shortening of the femoral neck is of increasing interest. Wang [9] discovered shortening within three months after surgery. We observed severe shortening of the femoral neck in some displaced fractures as early as one month after surgery. The occurrence of moderate and severe shortening reduced from 44.4 to 17.9% following FNS fixation with the pre-sliding technique to prevent displaced femoral neck fracture. Additionally, hip function one year postoperatively exhibited improvement compared to the traditional group. Yu and colleagues [19] hypothesize that femoral neck shortening may cause femoral head necrosis by finding that increasing degrees of femoral neck shortening resulted in higher peak acetabular stresses, uneven load distribution, and decreased hip mobility. Zlowodzki [11, 15, 20] discovered that individuals with femoral neck shortening of less than 5 mm had a greater likelihood of experiencing postoperative pain, claudication, and using crutches when compared to those with shortening of 5 mm or greater. The study on severe shortening revealed that 40% of the patients reported lower limb shortening while 30% used shoe inserts for augmentation.

### Indications for the use of pre-sliding

Displaced femoral neck fractures, cortical comminution, and poor reduction are among the risk factors for postoperative shortening. Displaced fractures of the femoral neck in young are frequently linked to high-energy traumas, often accompanied by cortical comminution injuries of the fractures, injuries to the hip joint’s capsule and ligaments, and reduced stability. The latter factor is one of the reasons for internal fixation failure [21].Previous literature has reported that 50–96% of femoral neck fractures are associated with cortical comminution of the fracture break, with 82% of cases located in the posterior medial column of the femoral neck [22]. The incidence of moderate and severe shortening of the femoral neck after fixation with the conventional method of FNS was observed to be 44.44%, which is similar to the data from previous studies [9, 21]. The possible mechanism is that FNS reserved sliding space of 20 mm exceeds the sliding distance required for typical fracture healing. Most clinical orthopedic surgeons now follow the guidelines and reserve a 20 mm distance, which is much greater than the required sliding distance, so anti-shortening is based on the reaction force that is generated by the cortical contact of the fracture, but displaced femoral neck fractures with cortical comminution in resulting in reduced contact area and anti-shortening. So for displaced femoral neck fractures, especially in combination with cortical comminution, we recommend the use of pre-sliding.

### Pre-sliding technique

FNS are manufactured in 5 mm units. If measurements are not in multiples of 5 mm, a shorter level of internal fixation must be chosen (e.g. if the initial measurement is 93 mm, then 90 mm should be selected). This will increase the TAD value. Additionally, pre-sliding can be utilized to choose a longer FNS, which is adjustable according to the length of the distance between the bolts and sliding grooves, with a maximum of 15 mm. Sliding grooves between 5 mm and 20 mm can be adjusted as needed. The use of pre-sliding technique reduced the median postoperative TAD value from 2.5 cm to 1.3 cm, similar to Cha [23] findings, the use of pre-sliding technique improves tip-apical distance (TAD) values by adjusting the depth of FNS bolts, and finite element analysis [24] demonstrates better stability with this technique compared to the traditional fixation method for Pauwels type III femoral neck fractures fixed with FNS. Our findings further confirm the efficacy of FNS pre-sliding technique in improving stability. After implementing the pre-sliding technique in FNS-fixed displaced femoral neck fractures, the femoral neck experienced less shortening than with the traditional method after 1 months post-surgery. Furthermore, there were no differences in fracture healing time or postoperative complications between the two groups. At the 1-year post-surgery mark, hip joint function was better. Since the sliding distance was restricted to only 5–10 mm, the maximum sliding space was limited, which enhanced the stability and anti-shortening of the FNS in displaced fractures. This improvement positively affects fracture healing by reducing unnecessary shortening and improving hip function.

### Precautions for FNS fixation of displaced femoral neck fractures

As a new internal fixation device for femoral neck fractures, FNS lacks validation from a prospective multicenter randomized cohort study, resulting in significant clinical uncertainty. Therefore, it is crucial to prevent early postoperative shortening. During the operation process for displaced femoral neck fractures, special attention should be given to this issue: ①Anatomical repositioning is an effective method to resist shortening by restoring the alignment relationship of the fracture and aligning the cortical ends of the bone to increase the contact area. ②Good preoperative planning should involve standardizing the operation according to the procedure and avoiding repeated operations to ensure the internal fixation is installed in the exact preoperative position. ③Use pre-sliding techniques to decrease the space for FNS sliding and pressurization, then choose an internal fixation device with a longer length based on the measurement results.

## Conclusion

In summary, after FNS fixation of displaced femoral neck fracture, there are cases of femoral neck shortening. This shortening negatively affects postoperative hip function. However, the use of the pre-sliding technique can reduce the occurrence of moderate to severe shortening, while simultaneously improving the postoperative TAD value. This technique does not affect fracture healing time and improves the postoperative hip function score at one year. The efficacy of FNS pre-sliding technique is satisfactory and deserving of wider adoption.

## Limitations

There were not enough poorly reset cases, they were not analyzed for poorly reset cases, and it was not possible to determine whether the pre-sliding technique could increase the anti-shortening effect in poorly reset cases. Inadequacies of this study include its retrospective cohort design with selection and recall bias, a small sample size, and a lack of comparison between nondisplaced femoral neck fracture and other fixation modalities. Therefore, it was not possible to determine the necessity of pre-sliding for nondisplaced femoral neck fracture and its impact on shortening incidence compared to other fixation modalities post-surgery. The follow-up period was brief, and longer monitoring is imperative to detect delayed complications such as femoral head necrosis.

## Data Availability

ALL data is provided within the manuscript.
